# All you can eat: is food supply unlimited in a colonially breeding bird?

**DOI:** 10.1002/ece3.1355

**Published:** 2015-01-02

**Authors:** Herbert Hoi, Ján Krištofík, Alžbeta Darolová

**Affiliations:** 1 Department of Integrative Biology and Evolution, Konrad Lorenz Institute of Ethology, University of Veterinary MedicineVienna, Austria; 2 Institute of Zoology, Slovak Academy of SciencesBratislava, Slovakia

**Keywords:** Bee-eater, colony, food surplus, *Merops apiaster*

## Abstract

Food availability is generally considered to determine breeding site selection and therefore plays an important role in hypotheses explaining the evolution of colony formation. Hypotheses trying to explain why birds join a colony usually assume that food is not limited, whereas those explaining variation in colony size suggest that food is under constraint. In this study, we investigate the composition and amount of food items not eaten by the nestlings and found in nest burrows of colonially nesting European bee-eaters (*Merops apiaster*). We aimed to determine whether this unconsumed food is an indicator of unlimited food supply, the result of mistakes during food transfer between parents and chicks or foraging selectivity of chicks. Therefore, we investigated the amount of dropped food for each nest in relation to reproductive performance and parameters reflecting parental quality. Our data suggest that parents carry more food to the nest than chicks can eat and, hence, food is not limited. This assumption is supported by the facts that there is a positive relationship between dropped food found in a nest and the number of fledglings, nestling age, and chick health condition and that the amount of dropped food is independent of colony size. There is variation in the amount of dropped food within colonies, suggesting that parent foraging efficiency may also be an important determinant. Pairs nesting in the center of a colony performed better than those nesting on the edge, which supports the assumption that quality differences between parents are important as well. However, dropped food cannot be used as an indicator of local food availability as (1) within-colony variation in dropped food is larger than between colony variation and, (2) the average amount of dropped food is not related to colony size.

## Introduction

Food availability is an important factor influencing an individual's reproductive success (Burger 1985; Wiens 1989; Ille and Hoi 1995; Davies and Deviche 2014; Herényi et al. 2014). Hence, abundance, distribution, and predictability of food are important determinants of breeding site selection (Lack 1968; Morse 1980; Gibbs et al. 1987; Brown et al. 1992; Brown and Brown 1996; Smith et al. 2007; Douglas et al. 2008; Van Klink et al. 2014). These food characteristics are consequently used to explain the evolution of coloniallity, in particular why animals join reproductive aggregations. The information-center hypothesis (Horn 1968; Ward and Zahavi 1973; Brown 1986; Greene 1987; Gori 1988) and the recruitment-center hypothesis (Richner and Heeb 1995, 1996; Kerth and Reckardt 2003) both assume that food is not a limiting resource and that joining a colony usually enhances the food accessibility for individuals (Brown et al. 2008). Alternatively, food availability can also be considered a constraint influencing competition over food and determining colony size (see Fretwell and Lucas 1970; Brown 1988; Shields et al. 1988; Griffin and Thomas 2000).

Explosive and ephemeral breeding events (salmon – Cunningham et al. 2013; Mowat et al. 2013; frogs – Grant et al. 2009), mast years (e.g., of European beech, *Fagus sylvatica* – Drobyshev et al. 2014), or cyclic outbreaks of insects (Hoi et al. 2004; Økland et al. 2005; Bonnot et al. 2009) provide a situation where food can be superabundant and lead to short-term foraging aggregations. There is still scarce empirical evidence for the importance of food for colonially breeding species (see Furness and Birkhead 1984). This is partly due to the fact that it is difficult to precisely determine food availability on the one hand and in relation to the number of consumers on the other hand (Brown and Brown 2001). The question arises how to prove whether food is, or is not, a limiting factor? One possibility to demonstrate a “land of milk and honey” situation would be to show that individuals of a species waste food by not consuming all prey captured while still developing optimally. Evidence for such a case of wasting food can be found in the colonial European bee-eater (*Merops apiaster*). It is known that complete prey items frequently remain unconsumed in their nest burrows, which are suggested to be dropped during the food transfer from parents to offspring (Cramp 1985; Horváth et al. 1992).

In this study, we therefore use the European bee-eater, a bird species breeding solitarily as well as in big colonies, as a model system to examine whether food which is dropped in the nest burrow and remains unconsumed is evidence for unlimited food availability. Hunting effort and handling effort together with its energetic content makes food normally a valuable item for consumption. Therefore, selection should enhance a careful handling of food items during transfer from parents to offspring. Only when food is unlimited and easy to hunt, selection pressure on a careful treatment of food might be relaxed and result in either accidently or actively dropped food items during food transfer, for example, because nestlings are full. Thus in support of the “unlimited food availability” hypothesis, we would expect that there is more food available than all members of a colony are able to use and adults may therefore quickly deliver more food than nestlings can eat. Consequently, we would predict that a certain portion of food will be wasted (dropped) in each pair of a colony. Furthermore, if food is in general unlimited and not just a local phenomenon, wasted (dropped), food should be found in all colonies and independent of the number of breeding pairs.

One basic assumption in relation to reproductive success is that wasting food should have no negative impact on offspring and adult birds. In contrast, one might predict that individuals in better condition may afford to waste more food. Thus, if food is wasted because there is more than enough for nestlings and adults, we would expect a positive relationship between reproductive success, in particular chick growth and the amount of food dropped. The amount of food dropped maybe also related to nutritional requirements. Assuming that nestlings waste more food the more often they are fed without suffering any costs, the amount of dropped food items should increase with nestling age or brood size.

We also explore alternative hypotheses to explain the occurrence of dropped food, for example, the “handling efficiency” hypothesis, which predicts that uneaten food could be simply related to the inability of nestlings to handle food. Less experienced and younger nestlings may make more mistakes when taking the food from the parents. Thus, we might predict a decrease in dropped food with chick age. The “nontasty food” hypothesis suggests that food delivered by the parents is less appropriate for nestlings, for example, too big, less tasty or venomous. In this context, we would predict a change with time in dropping items for some prey groups, for example, bumble bees. We would predict that older, more experienced chicks might be more selective and hence drop more food. The “constant drop rate” hypothesis assumes that prey are dropped accidently and hence at a constant proportion. Thus, we would predict that the amount of dropped food would increase with nestling number and age, but should be negatively related to reproductive success, nestling body condition, or health. Finally, dropped food items could be an indicator of colony quality. In line with this, we would predict a higher repeatability of dropped food items within than between colonies.

In order to investigate the role of unconsumed food as a signal for unlimited food supply, we examined the variation of dropped food within and between colonies and tested whether dropped food and/or its variance among nests are related to colony size. We further examined the relationship between dropped food and (1) nestling age and (2) breeding success in terms of number of fledglings, chick development, and health. To determine whether dropped food varies among individuals of a colony, for example, reflects variation in hunting efficiency of individuals, we additionally investigated the amount of dropped food for each nest in relation to (1) parent traits (morphological and conditional features), and (2) nest position in the colony (center vs. edge). To test the “nontasty food” hypothesis, we examine whether dropped food is a result of food being more or less tasteful or venomous. Therefore, we determined the proportion of different prey groups in relation to the age of chicks.

## Materials and Methods

### Study area and species

We conducted the investigation in southwestern Slovakia in the breeding season of 2009. European bee-eaters were studied at eleven sites within an area of 574 km^2^. The average (±SE) breeding density of bee-eaters was 4.5 ± 0.6 breeding pairs/km^2^. To determine colony size, we recorded the number of occupied nest burrows at each site. Data on the amount of dropped food in combination with clutch size, nestling numbers, and nesting success were collected from 26 pairs of 11 breeding sites including three solitary breeding pairs. Thus, we collected data from 1 to 5 nests of 11 breeding sites, in average 3 ± 0.46 (SE) pairs of eight sites, solitary breeding pairs not included. Nests were randomly selected from each colony. We further recorded morphological and serological measurements from nestlings of these 26 pairs. These 26 burrows were additionally classified according to the position in the breeding wall, namely at the center or the edge of a colony, as for most colonies nest burrows followed a linear horizontal distribution. Solitary burrows have been appointed to the edge category. In line with this, we determined 12 center and 14 edge burrows. Data on the amount of dropped food were collected for three sampling dates from 10 additional pairs. Thus, complete information on dropped food items is available for in total 36 nest burrows.

### Determining the amount of dropped food

**Figure 1:**
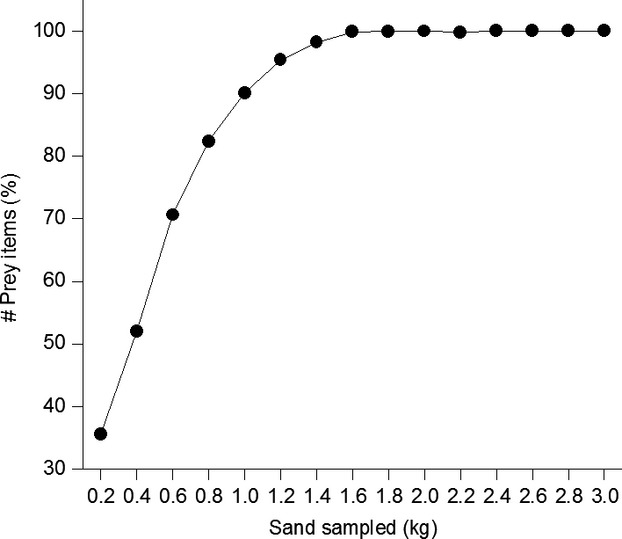
Relative frequency of prey items (insects) detected in successive sand samples (200 g each) taken from bee-eater burrows. Three kilogram of sand sampled.

Dropped food was collected on 6, 9, and 13 July 2009 from 36 nest burrows of 11 colonies. Samples have been collected between 10:00 a.m. and 15:00 p.m. (main feeding period, own observations). Weather conditions have been similar (hot and sunny) at the three sampling days. Bee-eater food, almost exclusively flying insects, is most active throughout the warmer daytime period. Thus, weather conditions and time should not affect the outcome of our study. By means of a spoon attached to a stick, about 2 kg of sand material was removed from each nest and stored in plastic bags. To estimate the accuracy of the sampling method, we took a total of 3 kg of sand material from additionally 12 randomly chosen nest burrows originating from six colony sites (two per site), collected the material in 200 g subsamples and stored them separately in numbered bags. Then, we recorded the number of prey items detected in each of the 200 g subsamples. As no more insects can be found after removing 2 kg of sand from the nest burrow (Fig.1), we assume that our sampling method accurately reflects the amount of surplus food in the nest burrow. Insects dropped in front of the entrance to a nest burrow were not included, because these prey items could be dropped by parent birds when being disturbed (own observations). We recorded the number of complete insects (not destroyed or partly eaten by the birds) and assigned them to eight different prey categories, namely Apiinae, Bombidae, Coleoptera, Hemiptera, Diptera, Orthoptera, Lepidoptera, and Odonata.

### Determining reproductive parameters

We measured breeding success for each nest using clutch size and chick survival for 15–20 days old nestlings. Nest inspections were conducted either by means of an endoscope or by counting the number of living or dead chicks or unhatched eggs, when removing chicks from the nest for taking morphological measurements and weight (see Hoi et al. 2002). We used a spoon tied to a pole (1.5 m in length) to remove the chicks from the nest. Nestlings of 26 nests were taken out of the nest burrow at the last sampling date (13 July 2009).

For each chick, we recorded wing length according to Svensson (1992) and weight by means of an electric balance to the nearest of 0.1 g. Chick condition was estimated for each nest. As it was impossible to accurately determine chick age for most nests, we first calculated the relationship between wing length (as a measure of size and chick age) and body weight. Wing length is considered to be a good predictor of nestling age in bee-eaters (Lessells et al. 1994). However, there is a marked weight recession in older chicks some days before they fledge. Therefore, to avoid heteroscedasticity and a nonlinear weight increase, we used only chicks within an approximate age range of four to 15 days (i.e., within 12 g at 4 days and 52 g at 15 days, Randík 1961; Belskaja 1976). The relation between wing length and body mass within this age range was already shown to be highly linear (Hoi et al. 2002). Weight deviation (residual weight) with wing length as the selection criterion (× variable) was used as a measure of chick condition, and mean residual nestling weight was calculated for each brood (*N *=* *26).

Adult birds were caught during the feeding period from 5 to 15 July by means of small claptraps attached to the entrance of the nest burrow. Adult birds were sexed on the basis of plumage characteristics including brightness of coloration and the pattern of the green lesser wing coverts (Glutz von Blotzheim and Bauer 1980; Cramp 1985; Hoi et al. 1998). We further recorded body weight (g) and measured wing length (in mm, according to Svensson 1992), and length of the sternum (including the carina until caudal end of metasternum) with the aid of a ruler. Blood samples, drawn from the brachial vein, were collected to estimate sedimentation rate and hematocrit. Hematocrit level is considered to be an indicator of general health status and an index of metabolic activity (Carpenter 1975; Gessaman et al. 1986; Harrison and Harrison 1986), and sedimentation rate increases in a wide range of infectious and inflammatory diseases due to an increase in blood circulating fibrinogen and *γ*-globulins (Gustafsson et al. 1994).

To measure sedimentation rate, the capillary tube was put into a refrigerated box (4°C) in an upright position for four hours. As sedimentation rate depends on proteins in the blood as well as on hematocrit, we regressed the sedimentation rate on hematocrit and used the residuals from this regression in the statistical analyses. To measure hematocrit, we centrifuged blood samples for 10 min at 1792 g and recorded hematocrit as the length of the tube containing erythrocytes.

In colonially breeding species, the centrality of a nest can be used as an estimator of parent quality (Brown and Brown 1996). To examine the effect of nest location for each colony, we classified edge and center nests see earlier.

### Statistical methods

Parametric tests were used throughout. To meet the assumptions for normality in some cases, data were log *x* + 1-transformed (mentioned in the text). To determine the importance of sample date (change over time) and colony origin for the number of food items dropped in the nest burrow in total, or for each insect category separately, we used a repeated measures ANOVA with the three successive sample dates as the repeated response factor and colony as the independent factor. To examine repeatability of dropped food in the three successive samples, we performed a repeatability analysis according to Lessells and Boag (1987).

A stepwise multiple regression analysis was used to examine possible relationships between reproductive parameters, adult quality measurements for males and females and the amount of dropped food. The analysis was run separately for males and females and for the following sets of independent variables: (1) morphological measurements including wing length, sternum length, and body weight, and (2) measurements of condition including residual body weight not explained by size (wing length), sedimentation rate, and hematocrit level. Statistical analyses are performed with the program SPSS 20.0.0 IBM Corp., Amonk, NY, USA.

## Results

### Is food remaining in the nest burrow surplus food?

**Figure 2:**
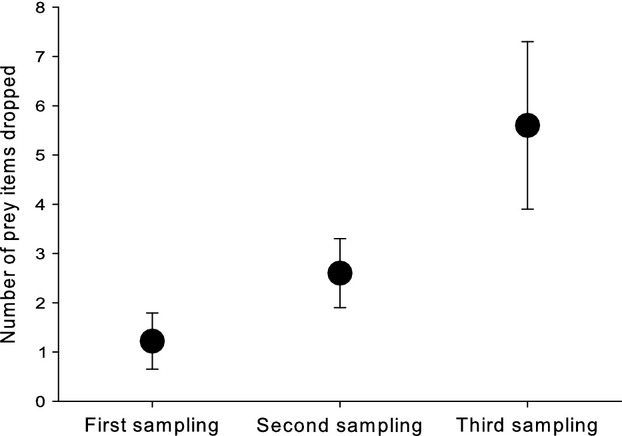
Increase of food items dropped with nestling age. Food items dropped (complete insects remaining in the nest burrow) sampled in bee-eater cavities initially (first sampling, 6 July), after three days (second sampling, 9 July), and after another four days (third sampling, 13 July). Given are means ± SE for 43 nests.

**Figure 3:**
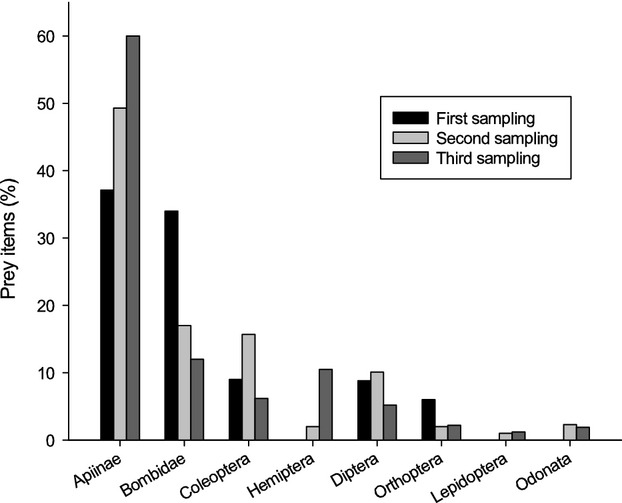
Composition of prey groups (%) in food items dropped for the three sampling dates.

The proportion of nests where we found dropped food items increased with sampling date (chick age) and was 37.8% (14 of 37) for the first, 61.1% (22 of 36) for the second, and 84.2% (32 of 38) for the third sampling. Also, the average amount of dropped food items in the nest significantly increased throughout the feeding period (Fig.2, Table1). The amount of food dropped in the nest burrow was in contrast independent of colony origin, and there was also no interaction between colony and sampling date (Table1). Consequently, we found a significant repeatability in the amount of food dropped in each nest burrow (*r *=* *0.57, df* *= 35, 72, *P *<* *0.0003, *F *=* *18.7), which was also significant for a subsample of 16 nests with a longer sampling interval of 10 days (*r *=* *0.74, df* *= 15, 16, *P *<* *0.002, *F *=* *16.8). Regarding single prey groups, we found a significant increase in only the number of bees and bugs (see Fig.3, Table1). Again colony origin and the interaction between colony origin and sample date have not been significant (Table1).

**Table 1:** Importance of sample date and colony origin for the number of food items dropped (in total, T), bees (Apiinae, AP) and bugs (Hemiptera, HP) dropped in the nest burrow based on a repeated measures ANOVA with food items dropped in each nest cavity at three successive sample dates as the repeated response factor and colony as the independent factor. *F* and *P*-values are given. Significant *P*-values are indicated in bold

	*F*			*P*			
	df	Ap	T	HP	T	AP	HP
Colony origin	10	0.24	0.31	0.59	0.98	>0.97	>0.79
Sample date	2	3.42	3.88	3.46	**0.041**	**=0.037**	**0.039**
Interaction	19	0.5	0.41	0.5	0.94	>0.9	0.94
**Total**	104						

### Is food dropped related to breeding success?

As mentioned above, there was an effect of nestling age. This is further supported by a stepwise multiple regression analysis. Chick age in terms of wing length, number of nestlings, and chick condition in terms of residual weight not explained by body size entered the regression model (*R*^2^ = 0.82, *F *=* *16.97, df = 3,24, *P *<* *0.0001). Partial correlation coefficients indicate a positive relationship between the number of food items dropped and the number of chicks (*r*_part_ = 0.81, *P *<* *0.0001), chick age (*r*_part_ = 0.74, *P *<* *0.0009), and chick condition (*r*_part_ = 0.57, *P *<* *0.01). Health parameters in terms of hematocrit level and sedimentation rate did not enter the model at a significance level of *P *=* *0.05.

**Figure 4:**
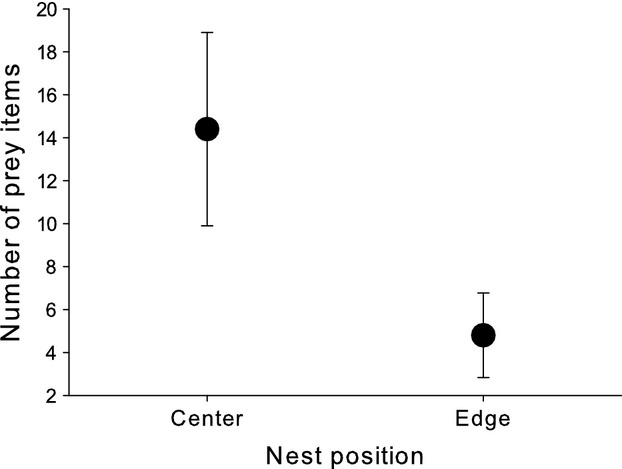
Food items dropped sampled in burrows of bee-eaters nesting in the center or on the edge of a colony.

**Figure 5:**
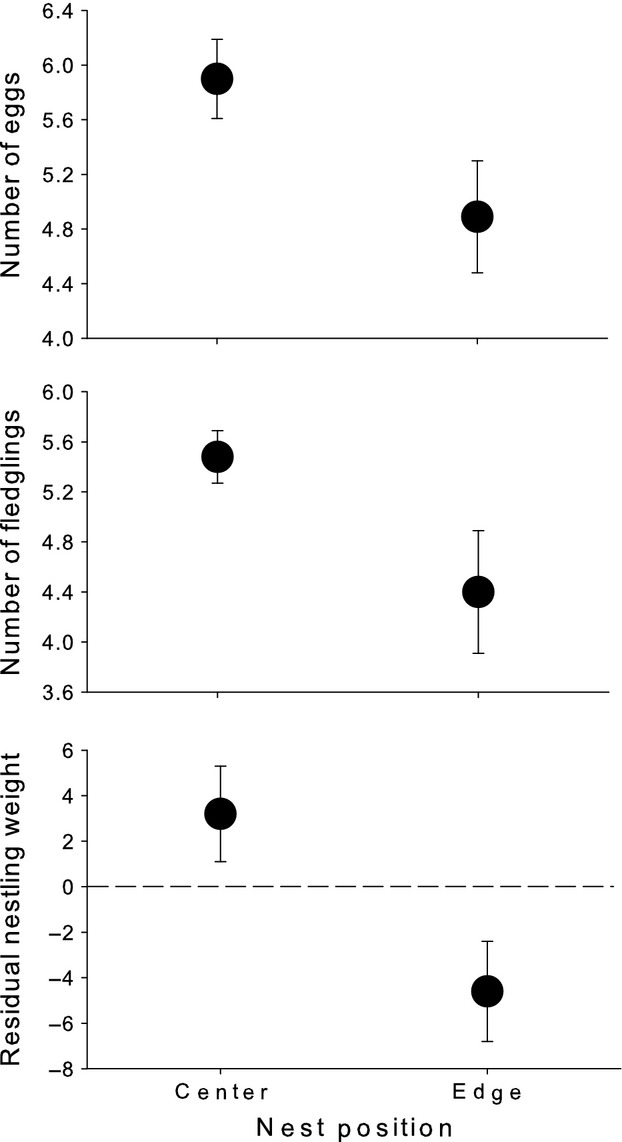
Clutch size (upper graph), number of fledglings (middle graph), and residual chick weight (body weight not explained by wing length) (lower graph) for bee-eaters nesting in the center or on the edge of a colony.

Center burrows contained significantly more dropped food items (paired *t*-test comparing nests: *t *=* *2.3, *P *=* *0.04, *N *=* *26; Fig.4). Birds nesting in center nests tended to lay more eggs (paired *t*-test: *t *=* *1.77, *P *=* *0.1, *N *=* *26; Fig.5) and fledge more chicks (paired *t*-test: *t *=* *3.8, *P *=* *0.001, *N *=* *26; Fig.5). In addition, chick development seems to be better in center nests (paired *t*-test: *t *=* *2.5, *P *=* *0.04, *N *=* *26; Fig.5).

### Is the amount of dropped food reflected in parent quality traits?

When running a stepwise multiple regression analysis separately for males and females, no morphological parameter or body weight entered the regression model (*P *>* *0.1). Examining conditional and serological measures, including body condition (residual body weight not explained by size), blood sedimentation rate, and hematocrit level, which might reflect quality and health status of adult bee-eaters in relation to dropped food, male hematocrit levels entered the regression model (*F *=* *5.94, *P *=* *0.028, df = 2,18, *R*^2^ = 0.28). The partial regression coefficient (*r*_part_ = 0.53) suggests a positive relationship between hematocrit levels of adult males and the amount of food dropped in the nest burrow. However, no variable entered the regression model when using the same variables for females.

### Does the amount of dropped food vary between breeding sites?

Examining the average amount of food items dropped between different breeding localities, we already mentioned that there is no effect of colony origin (see Table1). We further found no correlation between the number of breeding pairs/site and average number of food items dropped/site (*r *=* *0.03, *P *>* *0.9, *N *=* *11 localities). There is also no significant relationship between the variance of dropped food items within a colony and colony size (*r *=* *0.24, *P *>* *0.4, *N *=* *11). The frequency distribution for dropped food is, in general, right-tailed, which means that only a few pairs dropped a lot of food items. However, there was no relationship between skewness and colony size (*r *=* *−0.08, *P *>* *0.8, *N *=* *11).

## Discussion

According to our results, there is no evidence of food being a critical resource for colonial European bee-eaters at all. That food is unlimited rather than a constraint is indicated by the fact that we found unconsumed food items in almost every bee-eater nest burrow. In fact, up to 60 prey items can be found per nest and sample, which is quite a lot having in mind that these food items, not immediately eaten by nestling bee-eaters, are usually an important food source for commensal species living in the cavity. Fly and beetle larvae, which are very abundant in bee-eater holes (Krištofík et al. 1996; Petrescu and Adam 2001), can eat even big prey items completely (for instance, dragonflies, *Anisoptera* spp.) and within a few hours (own observations). Thus, the amount of prey dropped at a given moment does not accumulate over a long period. It rather reflects prey items dropped over a few hours up to half a day (own unpublished data). If food is limited, one would not expect that it would be wasted to such an extent. In fact, we are not aware of any other species where food transfer from parents to chicks results in such a high rate of lost food items. The assumption that food is easily accessible and not a constraint is further supported by the facts that the amount of unconsumed food in nest burrows (1) is independent of colony size, (2) is positively related to the number of fledglings per nest, (3) increases with chick age, and (4) is positively related to nestling condition. In contrast, hematocrit or sedimentation rates did not correlate with the amount of unconsumed food, which suggests that food availability has no direct effect on chick health.

Alternatively, as suggested by the “handling efficiency” hypothesis, one might argue that dropped food could simply be related to the ability of nestlings to handle food. Food can drop to the ground simply by mistake during the transfer from the parent to the nestling (Koenig 1959; Ursprung 1979; Helbig 1982). In this context, younger, less experienced chicks may be prone to make more mistakes when taking the food from the parents (Horváth et al. 1992). In this case, we would predict a decrease in the proportion of dropped food items with nestling age and experience. This argument is contradicted by our results showing that the number of food items dropped increases with nestling age. Precisely, we should use the proportion instead of the absolute number; however, the proportion of dropped food cannot be calculated in our study as we do not have data on age-dependent feeding rates.

Another explanation, suggested by the “nontasty food” hypothesis, could be that food delivered to the chicks is inappropriate for them, for instance, too big, less tasty, or venomous (e.g. stings of venomous insects not properly removed). This hypothesis is based on the assumption that nestlings are able to recognize these properties. Consequently, we would expect changes in the proportion of some prey groups, that is, bumble bees (*Bombus* spp.) in the unconsumed food in the course of time (experience, age of nestlings). Older, more experienced chicks might be more selective and hence drop more food because it is, for instance, less tasty or venomous. There is, in fact, a change in prey composition of food items dropped over time for some prey categories. However, there is no consistent change in venomous prey; for instance, bees appeared more frequently later on (Fig.3), whereas bumble bees seem to decrease (Fig.3). Only bugs show a significant change over time by increasing with time, which might indicate that older nestlings are more reluctant to eat them. However, bugs make up only 6.9% of unconsumed food, which suggests that they have no strong impact on the overall result. Bugs also constitute a small part of the adult diet (2.5% according to Krištín 1994). Nestling bee-eaters frequently eat noxious beetles (*Lytta vesicatoria*) and also malodorous bugs (*Aelia* spp*., Eurygaster* spp.) (Cramp 1985). Ursprung (1979) showed that the proportion of bugs increased in the diet of nestling bee-eaters with age, which contradicts the “nontasty” hypothesis. On the other hand, the two hypotheses are not mutually exclusive as there is no contradiction between selectivity and surplus food. Chicks can probably be more selective when food is in surplus.

A further explanation could be that unconsumed prey is dropped accidentally at a constant proportion and hence reflects varying feeding rates. In this way, the increase in unconsumed food with brood size and nestling age could be explained. However, such a high drop rate should be selected against especially when food is scarce (see also earlier); in fact, a waste of food is rarely documented in the animal kingdom.

One hypothesis discarded here is the handicap hypothesis (Zahavi and Zahavi 1997). If dropped food is wasted, it may in principal also signal a handicap as suggested by Zahavi and Zahavi (1997). In this context, the receiver of the signal would be the partner. However, food was dropped in the dark cavity and thus is unlikely to be detected easily.

Finally, if food dropped varies consistently between different colony sites, it could be used as an indicator of food availability at a given site. The high repeatability of unconsumed food in a nest suggests that it is a reliable measure as it is independent of variation over time and, hence, independent of changing weather conditions (Horváth et al. 1992). Although dropped food is highly repeatable for each nest, we found no difference between breeding localities, which suggests that dropped food does not appropriately reflect food availability for different breeding localities (colony sites). Consequently, we also found no relationship between colony size and food dropped. Thus, dropped food cannot be used as a measure of local resource availability. The occurrence of unconsumed food rather suggests a, in general, favorable food situation for European bee-eaters at the border of their breeding distribution (Glutz von Blotzheim and Bauer 1980; Cramp 1985).

On the one hand, we have shown that under normal circumstances, food is not a limiting resource in a bird species joining a colony which is theoretically predicted (Fretwell and Lucas 1970; Brown 1988; Shields et al. 1988; Griffin and Thomas 2000) but has not been proven for any colonial species up to now. On the other hand, parents bring more food to the nest than necessary and those occupying the center nests even seem to waste more prey captured. It seems likely that variation in parental quality adds to the observed variation in the amount of food brought to the nest. This is also supported by the right-tailed skew in the frequency distribution of dropped food, suggesting that some pairs produced large quantities, whereas the majority of pairs produced only smaller amounts; there were only a few pairs where we did not find any dropped food. The shape of this distribution as well as the within-colony variance of dropped food is independent of colony size; in contrast, the positive relationship in variance with colony size could be interpreted as an increase in the number (proportion) of pairs benefiting from bigger colonies. Thus, with regard to the foraging situation, the majority of breeding pairs is not constrained; however, a few pairs do very well, independent of colony size. Despotic behavior of early settling birds could be an explanation, as they may exclude others from the best foraging grounds (Fretwell and Lucas 1970; Ekman 1989). Phenotypic or intrinsic quality differences between individuals (e.g., age, experience or condition) could also be responsible for within-colony asymmetries and the obviously high variation in foraging and hunting efficiency (see also Brown and Brown 2001). In other words, an individual's intrinsic quality may determine the benefits when joining a breeding aggregation (Møller 1987; Brown and Brown 1996; Hoi and Hoi-Leitner 1997). Our results show a clear difference in the amount of food dropped between center and edge nests. However, it seems unlikely that the location in the colony directly affects the access to food sources (aerial insects). Nest location, rather, reflects settlement order and hence individual quality. Earlier arriving birds, which are usually older and more experienced (Brown and Brown 1996; Mitrus 2006; Vergara et al. 2007), probably settle in the center, whereas later arriving birds, probably those of lower quality, copy their habitat choice and settle around them (Danchin and Wagner 1997). This is also supported by the fact that pairs breeding in the center have an almost significantly higher clutch size (Fig.5) (see also Minias et al. 2011). We furthermore did not find a relationship between morphological or conditional parameters of parent birds. In the past, food was argued to be an important factor to explain the decline and sometimes even extinction of, in particular bird species, relying on large insect prey such as the European bee-eater (Reichhof 2014). Our results suggest now that actually food situation seems to be very favorable at the border of the distribution and probably other factors than food may limit further settlement and expansion attempts of the European bee-eater.
